# Predicting Performance in Working Memory During the Waking Period by Applying a Convolutional Neural Network to EEG Data in the N-Back Task: A Pilot Study

**DOI:** 10.3390/s26030772

**Published:** 2026-01-23

**Authors:** Masaya Shigemoto, Soma Shimizu, Kiyohisa Natsume

**Affiliations:** 1 Information Science and Technology Department, National Institute of Technology (KOSEN), Oshima College, Yamaguchi 742-2193, Japan; 2 Department of Human Intelligence Systems, Graduate School of Life Science and Systems Engineering, Kyushu Institute of Technology, Fukuoka 808-0196, Japan; natume@brain.kyutech.ac.jp

**Keywords:** circadian rhythm, convolutional neural network, EEG, N-back, working memory

## Abstract

Memory performance is regulated by circadian rhythms, and electroencephalograms (EEG) measure biological signals related to memory mechanisms and circadian rhythms. Therefore, EEG could be used to detect changes in diurnal memory. In this study, we measured the EEG signals of participants conducting a memory-related task and tested the effectiveness of a convolutional neural network (CNN) in predicting memory task performance at different times. EEG signals from participants performing N-back tasks at 8–9 a.m. and 3–4 p.m. were recorded. While performance showed no significant differences between times, differences were observed in EEG relative power. A CNN was trained using the relative power and raw waveform data of the EEG signals recorded during the tasks. When predicting the time at which the working memory (WM) was enhanced, the relative power CNN exhibited a significantly higher accuracy than the raw waveform CNN. However, the performance dropped in the test where the training data did not include the EEG data of the same participant. Overall, these results suggest that while EEG signals using a relative power CNN have high predictive potential, developing a personalized classification system that reflects individual chronotypes is effective for practical applications.

## 1. Introduction

Memory performance oscillates periodically because of circadian rhythms, with differences in memory performance being particularly pronounced when comparing day and night periods [1]. Studies in nocturnal rodents have reported that performance increases during the dark phase in spatial recognition memory tasks, such as the water maze [2], eight-arm radial maze [3], and novel object recognition tasks [4]. Furthermore, memory performance varies depending on the time of the day, even during the same activity phases. In mice, object recognition memory peaks during the early dark phase and exhibits significantly better performance than during the later dark phase [5]. Similarly, Drosophila exhibits a decrease in memory performance during the dark phase [6]. In humans, memory performance is regulated by circadian rhythms and is affected by other biological factors, such as sex and lifestyle. For example, working memory (WM), a short-term memory mechanism, decreases from 4–7 a.m. in female participants [7]. However, male participants showed no diurnal changes in WM function [8]. In addition, the peak time for WM performance differs depending on whether a person has a morning or evening lifestyle [9,10]. These results indicate that there are differences in diurnal changes in memory performance among individuals. Consequently, it is necessary to predict and propose an optimal time to achieve peak WM performance in a personalized manner.

In this study, we focused on electroencephalogram (EEG) readings as biological signals indicating memory performance. For example, among the different brain wave frequency bands, the theta (4–8 Hz) [11], alpha (8–12 Hz) [12], and beta1 (12–15 Hz) bands are involved in the construction of WM [13]. In addition, these memory-related EEG signals are modulated by circadian rhythms and changes in diurnal memory. Carbacol, an acetylcholine agonist, induces memory-related beta-wave-like oscillations in hippocampal slices [14] in vivo, and the frequency of these oscillations changes significantly during the night [15]. Recent studies have shown that deep learning methods are more effective for EEG classification than traditional machine learning algorithms, such as support vector machines and linear discriminant analysis [16]. Furthermore, the recording time zone can be predicted by learning the oscillation parameters of rat hippocampal slices using neural networks (NNs) [17]. It has also been reported that convolutional NNs (CNNs), which add a convolution layer to standard NNs, are more effective for human EEG classification [18,19,20] than recurrent NNs [21]. For example, CNN-based methods have been reported to be effective in epileptic seizure detection [22,23,24] and sleep stage classification [25,26,27]. Similarly, CNNs may enable the prediction of periods during which memory-related EEG activity is more likely to occur. Memory performance improves during chronotype-specific enhanced WM [28]. A CNN trained on EEG data during memory tasks can predict the time period in which task performance is enhanced, potentially suggesting optimal working hours for each individual. In the present study, we investigated diurnal changes in WM-related EEG and assessed the possibility of classifying diurnal EEG modulations using a CNN.

## 2. Materials and Methods

### 2.1. N-Back Task

**Figure 1:**
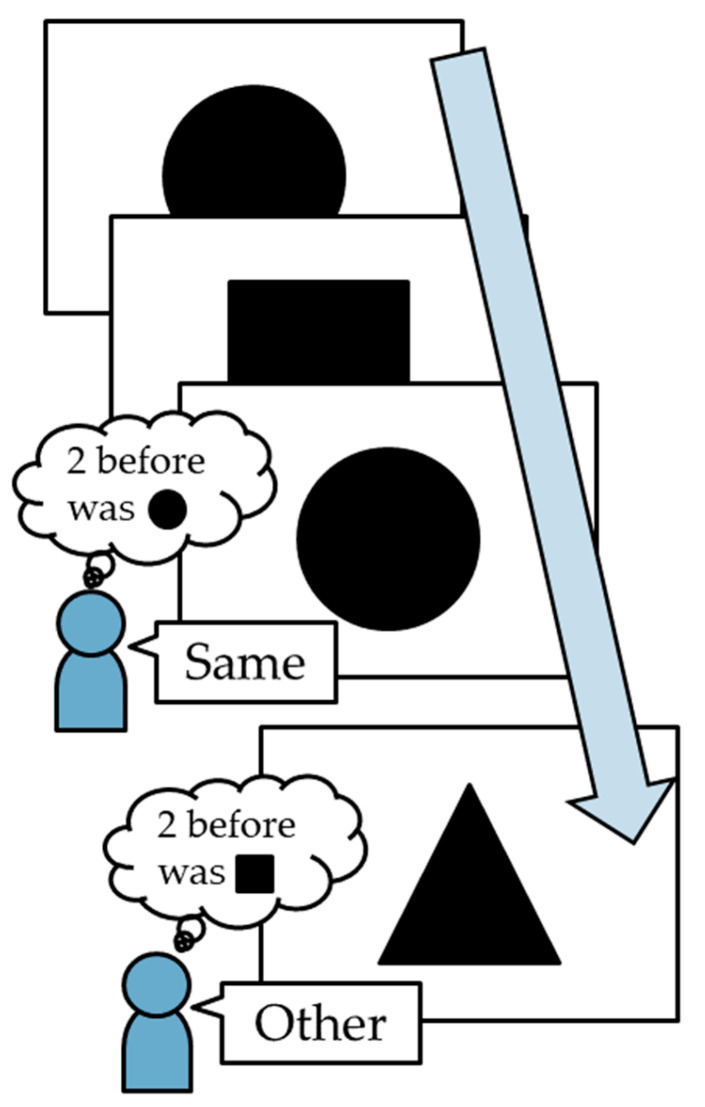
A schematic overview of the N-back task. The participant memorizes the displayed figure and answers whether the currently displayed figure is the “Same” or “Other” from the figure displayed two or three steps previously. The arrow indicates the sequence in which the shapes are displayed in the N-back task.

We used the N-back task to examine the participants’ WM performance. In this task, participants were required to memorize and compare the currently displayed stimuli with those presented N steps earlier [29,30]. The difficulty increased with an increase in N, and the correct answer rate consequently decreased [31]. The N-back task software was developed in Java 17 (Oracle Corporation, Austin, TX, USA), using the Eclipse 2022 (The Eclipse Foundation, Brussels, Belgium) integrated development environment. The software displayed one of the three shapes (square, circle, or triangle) on a computer screen with dimensions of 150 mm × 150 mm. The participant memorized the shape displayed on the screen and then indicated whether the newly displayed shape was the “Same” or “Other” compared to the N previous shapes (Figure 1). In this study, all participants performed the 2- and 3-back tasks twice at different times. The participants were instructed to answer 20 questions in each task as quickly as possible by clicking on a mouse. To investigate the WM performance, we calculated the correct answer rate (%) for each task.

The task was tested on five participants (three males and two females; all aged 19–20 years), who provided written informed consent to participate and had eaten breakfast on the day of the experiment. All participants provided informed consent before participating in the experiment. The experiments were approved by The Kyushu Institute of Technology Human Experimental Committee (#23-04) and the Human Research Ethics Committee of the National Institute of Technology (KOSEN), Oshima College.

To investigate whether changes in memory-related brain waves within the same waking period and active phase could be classified, we tested the participants in both the morning (8–9 a.m.) and afternoon (3–4 p.m.) on the same day. After the participants were informed of the rules of the N-back task, they were fitted with an EEG device to begin the task. All participants practiced the 2- or 3-back task once, then performed the same task twice to measure the correct answer rate and EEG signals. We compared the relative powers between the high- and low-scoring groups, which were classified by trial and showed the highest and lowest correct answer rates in the N-back task, respectively, for all participants. If the tests had the same answer rates, we selected the sessions with the shortest and longest response times as the high and low scores, respectively, because the response time of the N-back task was used to evaluate WM performance [32].

### 2.2. EEG Recording

**Figure 2:**
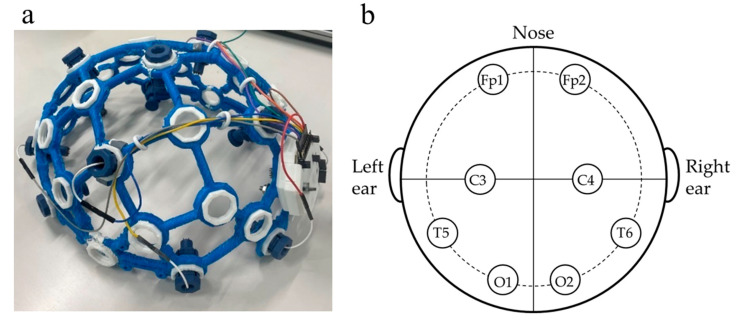
(**a**) The electroencephalograph in the experiment comprising an OpenBCI™ 8-channel board and frame. (**b**) Diagram of the EEG measurement locations Fp1, Fp2, C3, C4, T5, T6, O1, and O2.

EEG measurements were conducted using the OpenBCI™ 8-channel Cyton Biosensing Board (OpenBCI, New York, NY, USA), an open platform for EEG measurements [33]. The electroencephalograph frame was printed using a 3D printer (MF-2200D; MUTOH INDUSTRIES Ltd., Tokyo, Japan) with a TPU filament as the material (Figure 2a) and units containing electrodes at eight locations (Fp1, Fp2, C3, C4, T5, T6, O1, and O2) according to the international 10–20 system (Figure 2b). Signals were recorded at a sampling frequency of 250 Hz using OpenBCI_GUI v6.0.0-beta.1 EEG recording software (OpenBCI, New York, NY, USA). The recorded EEG signals were output in TXT format using OpenBCI_GUI, and all signals were analyzed using a program developed in Python 3.13.7 (Python Software Foundation, Wilmington, DE, USA) using the MNE library. All signals were normalized to adopt a mean of 0 and a variance of 1, after which a bandpass filter was applied, passing from 2 to 50 Hz. Each signal was subjected to independent component analysis (ICA) [34,35] to remove signal noise, and the time window containing the blink waveform was visually cut off.

The EEG recording was initiated when the first figure was displayed, and it ended when the participants answered the last question. The measured EEG data were fast Fourier transformed to obtain the power values for each frequency band: delta (2–4 Hz), theta (4–8 Hz), alpha (8–12 Hz), beta1 (12–15 Hz), beta2 (15–30 Hz), and gamma (30–50 Hz). The relative frequency powers were calculated by dividing the power value of each frequency band by the sum of all the band values. The relative powers were calculated from the signals from 5 to 15 s after the start of each trial and were used to compare the EEGs.

### 2.3. CNN Structure

**Figure 3:**
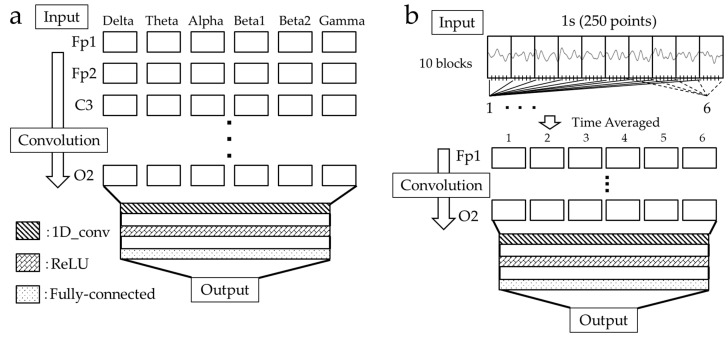
Configuration diagram of the CNN. (**a**) The CNN of the relative power comprises an input layer, 1D convolution layer (1D_conv), fully connected layer, and output layer. (**b**) The CNN of the raw waveform comprises an input layer, 1D convolution layer (1D_conv), ReLU, fully connected layer, and output layer, which were subsequently converted into six dimensions. The raw waveform in the input layer was divided into 10 blocks, and each block was subdivided into six time-segments.

In this study, we used the relative power (six groups) and raw waveform for 1 s (250 points) of the signal during the N-back task to train the CNN and compare its accuracy. The CNN was developed in Python 3.13.7 using the TensorFlow library. The CNN comprised an input layer, convolution layers, fully connected layers, and an output layer (Figure 3a). To enable comparison with the relative power, the raw waveform data were time-averaged and converted into six dimensions for training. The 250-point waveform was initially divided into ten blocks (25 points each). Each block was subdivided into six time-segments (4–5 points). Finally, the time segments were averaged across all ten blocks to produce six-dimensional data (Figure 3b). The data were convolved in the channel direction using a one-dimensional (1D) convolution layer (1D_conv) consisting of 128 filters with a kernel size of 1. A rectified linear unit (ReLU) function was applied to the output of the convolution layer. Subsequently, the data were passed to a fully connected layer with 32 units, and a softmax function was applied for classification.

To calculate the accuracy rate defined in Equation (1), we initially determined the numbers of correctly classified positive cases (true positives, TP), correctly classified negative cases (true negatives, TN), incorrectly classified negative cases (false positives, FP), and incorrectly classified positive cases (false negatives, FN).

Accuracy rate = (TP + TN)/(TP + TN + FP + FN)(1)

The CNN was trained using 1-s raw waveform data during the N-back task in both the a.m. and p.m. sessions from all of the participants’ trials. Raw EEG waveforms were cropped by sliding the time window by 0.1 s from 3 to 15 s from the start of the N-back task. Six different relative powers were calculated for all raw waveforms and used as training data. These data were classified into training and testing sets in a 70:30 ratio, with the proportion of the validation dataset at 30%, with 1000 epochs, and a batch size of 128. The training data were randomly selected ten times, and the accuracy rates were averaged. We conducted leave-one-out cross-validation (LOOCV) to verify the accuracy of the model without relying on subject-specific patterns. During this process, data from one participant were excluded from the training set and used exclusively for testing. This procedure was repeated for all participants to ensure generalizability of the model.

We trained the CNN in two classes with two patterns (a.m. versus p.m. and high- versus low-score) of EEG data and tested whether they could be classified correctly.

### 2.4. Statistical Analysis

Owing to the small sample size, we used exact nonparametric tests instead of asymptotic approximations. We performed the exact Wilcoxon signed-rank test for paired comparisons and exact Wilcoxon rank-sum test for independent comparisons.

Considering the exploratory nature of this pilot study, no correction for multiple comparisons was applied to avoid increasing the risk of Type II errors (false negatives). Therefore, the *p*-values reported in this study should be interpreted as preliminary.

Data are expressed as the mean ± standard error of the mean (SEM). Statistical significance was set at *p* < 0.05.

## 3. Results

### 3.1. Diurnal Change in WM Performance

**Figure 4:**
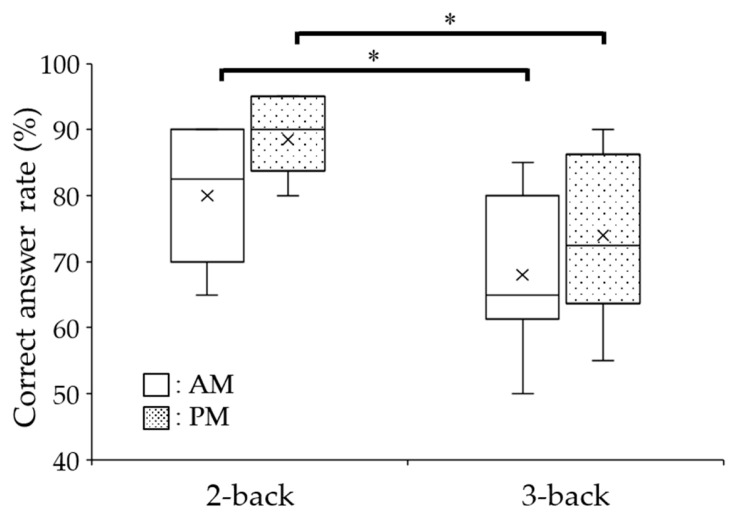
Comparison of the participants’ N-back task performances between a.m. and p.m. Data are shown as box plots depicting the correct answer rates at a.m. and p.m. in both the 2- and 3-back tasks. The cross mark indicates the mean value. Wilcoxon signed-rank test; * *p* < 0.05.

A comparison of the correct answer rates between the 2- and 3-back tasks revealed significant differences between the a.m. and p.m. experiments (Wilcoxon signed-rank test, a.m.: *p* = 0.04; p.m.: *p* = 0.02) (Figure 4). Next, we compared the average correct answer rates of the a.m. and p.m. experiments. There were no significant differences in the correct answer rates between a.m. and p.m. for the 2- (88.5 ± 1.8% for a.m. versus 80.0 ± 3.0% for p.m.; Wilcoxon signed-rank test, *p* = 0.13) or 3-back task (68.0 ± 3.8% at a.m. versus 74.0 ± 4.1% at p.m.; Wilcoxon signed-rank test, *p* = 0.25). When we classified the high- and low-score results based on the correct answer rate, all participants showed a high score for p.m. in the 2-back task and a high score for both a.m. and p.m. in the 3-back task (Table 1).

### 3.2. EEG Relative Power

**Figure 5:**
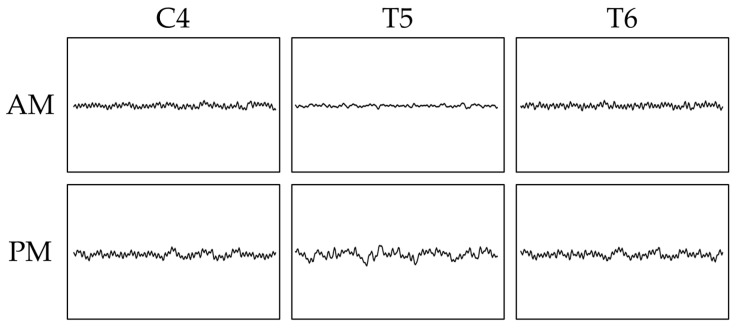
The waveforms of the channels showing significant differences between the a.m. and p.m.

We calculated the relative powers for all trials for each participant and compared the measures for both a.m. and p.m. (Table 2). In the 2-back task, the theta rate of T6 was significantly higher at p.m. (35.4 ± 3.2%) than at a.m. (24.1 ± 2.2%) (Wilcoxon signed-rank test, *p* = 0.01). Conversely, in the 3-back task, the delta rate of C4 was significantly higher at a.m. (2.0 ± 0.4%) than at p.m. (1.1 ± 0.1%) (Wilcoxon signed-rank test, *p* = 0.02), and the delta rate of T5 was also higher at a.m. (2.0 ± 0.4%) than at p.m. (0.9 ± 0.2%) (Wilcoxon signed-rank test, *p* = 0.01) (Figure 5).

Next, we compared EEG signals between the high- and low-scoring groups (Table 3). In the 2-back task, none of the relative powers were significantly different at any location (Wilcoxon signed-rank test, *p* > 0.05). In addition, none of the relative powers were significantly different at any location in the 3-back task (Wilcoxon signed-rank test, *p* > 0.05). However, the delta rate at T5 tended to be higher in the high-scoring group on the 3-back task (Wilcoxon signed-rank test, *p* = 0.06).

### 3.3. Prediction of Time Zones Using CNN

**Figure 6:**
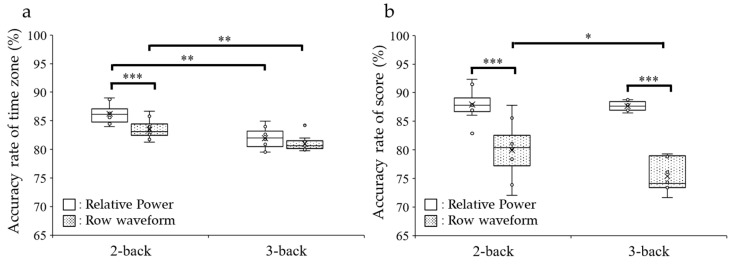
Predictions using CNN. (**a**) Box plots depicting the accuracy rates of the measured time zone of 2- and 3-back tasks using relative power and raw-waveform parameters of the test. (**b**) Box plots depicting accuracy rates of score of 2- and 3-back task using relative power and raw-waveform of test. Wilcoxon rank-sum test; * *p* < 0.05, ** *p* < 0.01, *** *p* < 0.001.

Subsequently, we investigated the ability of the CNN to predict the measured time zone of the N-back task using EEG data (Figure 6a). First, the relative power parameter was used to train the CNN. In the 2-back task, the accuracy rate was 86.7 ± 0.2% for the training data and 86.2 ± 0.5% for the test data. In the 3-back task, the accuracy rate was 82.8 ± 0.4% for the training and 82.0 ± 0.6% for the test, and it was significantly lower than the results of test data for the 2-back task (Wilcoxon rank-sum test, *p* < 0.001).

Subsequently, an EEG raw waveform was used to train the model to predict whether the tests were conducted in the a.m. or p.m. In the 2-back task, the accuracy rate was 83.1 ± 0.4% for training and 83.6 ± 0.5% for the test data, which was significantly lower than the relative power (comparison of results at test; Wilcoxon rank-sum test, *p* = 0.002). In the 3-back task, the accuracy rate was 81.5 ± 0.4% for training and 81.0 ± 0.4% for test data, which was also significantly lower than the relative power (comparison of results at test; Wilcoxon rank-sum test, *p* = 0.005).

The generalization capability of the time zone CNN was evaluated using the LOOCV method to exclude subject-specific bias (Table 4). For relative power in both the 2- and 3-back tasks, accuracies fell to near-chance levels (approximately 50%), underperforming the standard validation, and the subject data were included. The performance of the raw waveforms also degraded substantially. In particular, Participant 5 showed the lowest performance, with an accuracy of 18.6 ± 1.8% in the 2-back task.

### 3.4. Prediction of the N-Back Score Using CNN

Next, we investigated the ability of the CNN to predict high or low scores on the N-back task using EEG signals (Figure 6b). Using the relative power in the 2-back task, the accuracy rate was 89.4 ± 0.5% for the training data and 87.9 ± 0.8% for the test data, showing no significant difference compared to the prediction of the time zone (Wilcoxon rank-sum test, *p* = 0.09). In the 3-back task, the accuracy rate was 89.3 ± 0.2% for the training and 87.7 ± 0.3% for the test. There was no significant difference in the accuracy of the relative power CNN for the 2- and 3-back tasks (Wilcoxon rank-sum test, *p* = 0.22). In contrast to the 2-back task, the accuracy rate was significantly higher than that of the time-zone prediction (Wilcoxon rank-sum test, *p* = 0.02).

Next, we used the raw waveform for training to predict the high or low scores. In the 2-back task, the accuracy rate was 82.8 ± 0.5% for training and 80.0 ± 1.5% for test data, which was significantly lower than the relative power (Wilcoxon rank-sum test, *p* < 0. 001). In the 3-back task, the accuracy rate was 77.1 ± 0.5% for training and 75.4 ± 0.9% for test data, significantly lower than the relative power (Wilcoxon rank-sum test *p* < 0.001) and 2-back task’s raw-waveform (Wilcoxon rank-sum test *p* = 0.03). Similar to the relative power, the accuracy rate was significantly higher than that for the prediction of the time zone in the 3-back task (Wilcoxon rank-sum test, *p* < 0.001).

Similar to the time zone classification, the score classification was evaluated using LOOCV (Table 5). Consequently, regardless of whether relative power or raw waveforms were used, the accuracy in both the 2- and 3-back tasks dropped to near-chance levels, or approximately 30%.

## 4. Discussion

### 4.1. Modulation of Working Memory-Related EEG

To investigate whether memory-related EEG changes occurred within the same activity period, we compared EEG signals during the N-back task in the a.m. (8–9 a.m.) and p.m. (3–4 p.m.) on the same day. First, we compared WM performance between the a.m. and p.m. groups using an N-back task with five participants each. This analysis revealed no significant differences in the correct answer rates between the a.m. and p.m. groups in either the 2- or 3-back tasks. In contrast to the correct answer rate, the relative power of EEG signals showed diurnal changes. The theta rate at T5 increased significantly in the p.m. during the 2-back task. Prior research has shown that theta waves are important for synchronization between serial brain regions in working memory [11,36]. In addition, the occipital area, including T5, is close to the visual cortex and involved in the processing of visual information [37,38]. Consequently, the memory consolidation of visual information is more likely to increase in the p.m., although this is not sufficient to affect the correct answer rate. There are individual variations in diurnal changes in WM tasks owing to differences in lifestyle, such as morning or evening types [9,10]. However, as this experiment was conducted with five students of similar age from the same school, it is considered that the typical diurnal changes in brain waves for the morning types were recorded. In the 3-back task, the EEG showed significantly lower delta rates in the C4 and T5 regions of the a.m. Delta power in the occipital lobe increases as the cognitive task becomes more difficult [39,40]; therefore, it is possible that WM is loaded more on the participants in the a.m.

We compared the EEGs signals of each participant by classifying them into high- and low-scoring groups based on the correct answer rate. In the 3-back task, high-scoring EEGs tended to have higher T5 deltas than low-scoring EEGs. As previously described, delta power in the occipital lobe increases with increasing white matter load [39,40]. Consequently, an increased load may have been necessary to accurately accumulate visual memory during the 3-back task, leading to the observed increase in delta power. Differences in task difficulty revealed distinct patterns, whether classified by the time zone or score. Classifying the time periods that enhance memory performance according to the degree of working memory load is crucial.

### 4.2. Classification of Working Memory-Related EEG Using CNN

In the present study, we classified the relative power during the 2-back task for the a.m. and p.m. classes using a CNN, finding an accuracy rate of 86.2 ± 0.5% for the test data. It has previously been reported that the classification accuracy increases with the integrated channel information of EEGs for NNs [41,42]. Similarly, convolution in the channel direction showed a higher accuracy rate despite the small number of parameters in the input layer. Because classification using neural networks based on EEG frequency band ratios has been previously reported, we investigated the accuracy rates of the band ratios. For the relative power of the 3-back task, the accuracy rate was 82.0 ± 0.6%, which was significantly lower than that of the 2-back task. This indicates that an increased memory load may make time-zone classification more difficult. Therefore, we used the time-averaged raw waveforms to train the CNN and compared them with the band and accuracy rates. The test showed an accuracy rate of 83.5 ± 0.5% for the 2-back task and 81.0 ± 0.4% for the 3-back task, both significantly lower than the relative power. It is likely that convolution in the temporal direction in learning the relative powers enabled classification with higher accuracy, similar to other studies that used CNNs to classify EEG signals [22,25,26,43]. Even with the limited dimensionality of six-dimensional data, known as relative power, channel-wise convolution remains effective.

We subsequently classified the high- and low-score EEG signals using the CNN, and the results showed high accuracy rate in both cases of relative power: 87.7 ± 0.6% for the 2-back and 88.7 ± 0.6% for the 3-back task. In addition, the accuracy rates were significantly higher than those of the time-zone CNN for both tasks, although there was no significant difference in the EEG relative power between high and low scores. Similar to the findings of the present study, no significant changes in the EEGs were observed for tasks with excessive working memory loads, such as the 3-back task [44]. The CNN using relative power achieved high accuracy in predicting scores, even for the 3-back task, in which no significant difference was observed between the a.m. and p.m. sessions. Cognitive processes, including WM, involve large-scale functional networks distributed across the brain [45,46]. While evaluating isolated channels may fail to capture the holistic neural state, the CNN can overcome this limitation by integrating information across multiple channels through its convolutional layers.

Finally, a limitation of this study is that the participants’ chronotypes were not assessed. Indeed, the model demonstrated poor generalization to novel participants, as evidenced by the low LOOCV scores. This is likely due to the high degree of inter-participant variability in EEG signals. In particular, cases in which accuracy dropped below chance levels (e.g., Participant 5′s time-of-day classification in the 2-back task) imply that different chronotypes may exhibit contrasting circadian patterns of WM. Consequently, to implement this system in society, an approach based on individualized calibration, adjusting for each user’s chronotype, would be a more viable and effective strategy.

## 5. Conclusions

In this study, we investigated the possibility of predicting memory performance times during the same activity phase using EEG. By training a CNN on the EEG relative powers, we could predict the time zone in which a memory task was conducted. Furthermore, EEG relative power enabled the prediction of whether the memory experiment results would be high- or low-scoring. However, the LOOCV scores were low, indicating that the ability of the model to generalize to novel participants was limited. In the future, it will be necessary to measure the EEG signals of various participants with various chronotypes and classify them using CNNs. The various chronotypes necessitate the proposal of a method for estimating the circadian rhythm of memory by combining EEG data with circadian rhythm measurement devices, such as actigraphs [47] and smartwatches [48].

## Figures and Tables

**Table 1:** Correct answer rates for all participants. Bolded scores indicate the high score, and underlined scores indicate the low score.

2-Back	a.m.		p.m.		3-Back	a.m.		p.m.	
	1st	2nd	1st	2nd		1st	2nd	1st	2nd
Participant 1	75%	70%	80%	**90%**		65%	**80%**	60%	70%
Participant 2	90%	90%	85%	**90%**		75%	85%	85%	**90%**
Participant 3	85%	65%	**95%**	85%		50%	**80%**	75%	65%
Participant 4	80%	70%	80%	**95%**		65%	65%	**90%**	85%
Participant 5	90%	85%	90%	**95%**		50%	65%	55%	**65%**

**Table 2:** Relative powers in the a.m. and p.m.

	Delta			Theta			Alpha			Beta1			Beta2			Gamma		
2-Back	a.m.	p.m.	*p*	a.m.	p.m.	*p*	a.m.	p.m.	*p*	a.m.	p.m.	*p*	a.m.	p.m.	*p*	a.m.	p.m.	*p*
Fp1	1.4 ± 0.2	1.2 ± 0.2	0.31	38.0 ± 4.1	41.0 ± 2.7	0.38	26.8 ± 3.5	27.0 ± 1.3	0.30	16.8 ± 1.7	14.8 ± 1.2	0.30	10.9 ± 1.6	10.3 ± 1.3	0.92	6.1 ± 0.4	5.6 ± 1.0	0.64
Fp2	1.3 ± 0.3	1.1 ± 0.2	1.00	35.5 ± 4.3	41.2 ± 3.3	0.63	27.1 ± 4.3	24.7 ± 2.2	0.85	15.0 ± 1.1	13.8 ± 1.2	0.63	13.3 ± 2.3	12.0 ± 2.6	0.82	7.8 ± 1.0	7.2 ± 1.7	0.77
C3	1.2 ± 0.2	1.0 ± 0.2	0.47	34.4 ± 2.4	41.7 ± 2.8	0.11	30.4 ± 3.2	27.3 ± 1.6	0.65	16.3 ± 1.3	15.5 ± 1.1	0.84	11.0 ± 1.2	9.2 ± 0.7	0.43	6.7 ± 0.5	5.3 ± 0.7	0.19
C4	1.4 ± 0.2	1.1 ± 0.2	0.30	39.4 ± 2.8	37.2 ± 3.0	0.65	30.3 ± 2.0	29.9 ± 2.0	0.92	15.2 ± 1.6	15.0 ± 0.8	0.91	8.4 ± 0.7	10.6 ± 1.3	0.25	5.3 ± 0.5	6.2 ± 1.0	0.63
T5	1.5 ± 0.2	0.8 ± 0.2	0.03	37.3 ± 4.3	34.4 ± 3.5	0.70	27.1 ± 3.7	27.5 ± 0.8	0.63	17.8 ± 2.4	17.3 ± 1.4	0.84	10.4 ± 1.4	12.3 ± 1.3	0.56	5.9 ± 0.6	7.7 ± 1.3	0.50
T6	1.2 ± 0.6	0.6 ± 0.1	1.00	24.1 ± 2.2	35.4 ± 3.2	**0.01**	29.0 ± 2.3	29.2 ± 1.1	1.00	20.4 ± 1.5	18.2 ± 1.6	0.43	16.1 ± 2.5	10.6 ± 1.9	0.08	9.1 ± 1.5	5.9 ± 0.9	0.06
O1	0.9 ± 0.2	0.8 ± 0.2	0.46	24.6 ± 2.7	33.6 ± 4.5	0.28	28.2 ± 3.3	24.5 ± 1.6	0.73	21.3 ± 1.9	18.2 ± 1.8	0.19	15.3 ± 2.1	14.1 ± 2.3	0.70	9.7 ± 1.4	8.8 ± 2.0	0.70
O2	1.0 ± 0.2	1.0 ± 0.3	0.94	30.6 ± 2.8	34.9 ± 5.1	0.56	26.0 ± 2.3	26.3 ± 1.6	0.85	19.9 ± 1.6	17.5 ± 1.7	0.23	14.4 ± 1.4	12.8 ± 2.5	0.50	8.1 ± 0.9	7.6 ± 1.5	0.77
	**Delta**			**Theta**			**Alpha**			**Beta1**			**Beta2**			**Gamma**		
**3-Back**	**a.m.**	**p.m.**	***p***	**a.m.**	**p.m.**	***p***	**a.m.**	**p.m.**	***p***	**a.m.**	**p.m.**	***p***	**a.m.**	**p.m.**	***p***	**a.m.**	**p.m.**	***p***
Fp1	2.3 ± 0.4	1.7 ± 0.3	0.30	44.1 ± 2.9	40.8 ± 3.8	0.63	22.9 ± 1.5	22.6 ± 0.9	1.00	14.5 ± 1.5	15.2 ± 1.4	0.81	10.2 ± 1.3	13.1 ± 1.8	0.43	6.0 ± 0.7	6.6 ± 0.6	0.77
Fp2	2.3 ± 0.3	1.3 ± 0.2	0.11	41.7 ± 3.4	39.0 ± 3.9	0.63	22.4 ± 2.7	23.1 ± 1.4	0.43	13.6 ± 1.1	16.7 ± 1.6	0.19	12.1 ± 1.9	12.2 ± 1.8	0.77	7.9 ± 1.4	7.7 ± 1.0	0.70
C3	1.9 ± 0.3	1.2 ± 0.3	0.38	42.2 ± 2.4	42.1 ± 2.6	1.00	24.8 ± 1.6	25.5 ± 1.5	0.65	16.2 ± 1.4	15.5 ± 0.9	0.70	9.6 ± 0.9	9.6 ± 0.7	0.91	5.2 ± 0.5	6.1 ± 0.7	0.15
C4	2.0 ± 0.4	1.1 ± 0.1	**0.02**	42.7 ± 2.9	39.2 ± 2.7	0.56	25.6 ± 1.5	28.1 ± 1.2	0.30	15.5 ± 1.4	15.8 ± 1.3	0.64	9.0 ± 1.0	10.3 ± 0.9	0.25	5.2 ± 0.4	5.5 ± 0.5	0.55
T5	2.0 ± 0.4	0.9 ± 0.2	**0.01**	39.0 ± 3.8	35.3 ± 3.5	0.32	23.5 ± 2.2	24.3 ± 1.0	1.00	16.9 ± 1.5	17.7 ± 1.2	0.63	12.0 ± 1.8	13.3 ± 1.4	0.49	6.5 ± 0.6	8.5 ± 1.0	0.07
T6	1.1 ± 0.3	0.8 ± 0.1	0.88	30.2 ± 3.2	35.5 ± 3.4	0.28	24.6 ± 1.5	26.8 ± 1.2	0.28	20.0 ± 1.2	19.0 ± 1.4	0.58	16.1 ± 2.4	11.7 ± 1.1	0.15	8.0 ± 1.5	6.1 ± 0.6	0.38
O1	1.1 ± 0.2	0.8 ± 0.1	0.22	29.7 ± 2.3	28.2 ± 4.5	0.70	25.6 ± 2.9	24.5 ± 2.5	0.85	19.8 ± 1.2	21.7 ± 2.0	0.56	14.6 ± 1.8	15.3 ± 2.9	0.92	9.2 ± 1.2	9.5 ± 2.4	1.00
O2	1.4 ± 0.3	1.0 ± 0.3	0.55	36.7 ± 3.6	37.2 ± 4.8	0.77	23.1 ± 1.7	25.5 ± 1.4	0.38	18.8 ± 2.0	16.6 ± 2.2	0.31	12.9 ± 2.2	12.1 ± 2.0	1.00	7.1 ± 1.2	7.5 ± 1.1	0.77

All *p*-values were calculated using the Wilcoxon signed-rank test, and bold values indicate *p* < 0.05.

**Table 3:** Relative powers of the high and low scores.

	Delta			Theta			Alpha			Beta1			Beta2			Gamma		
2-Back	High Score	Low Score	*p*	High Score	Low Score	*p*	High Score	Low Score	*p*	High Score	Low Score	*p*	High Score	Low Score	*p*	High Score	Low Score	*p*
Fp1	1.4 ± 0.2	1.1 ± 0.2	0.50	39.9 ± 3.8	38.8 ± 4.2	0.63	25.1 ± 2.4	27.3 ± 1.4	0.19	17.7 ± 2.0	14.2 ± 1.7	0.25	10.8 ± 2.7	11.4 ± 2.4	0.25	5.1 ± 0.9	7.3 ± 1.6	0.19
Fp2	1.2 ± 0.3	1.1 ± 0.1	0.75	38.9 ± 4.4	40.5 ± 6.7	1.00	24.7 ± 3.5	22.4 ± 2.5	0.13	15.4 ± 0.7	13.6 ± 2.0	0.63	13.1 ± 3.7	13.8 ± 4.9	0.88	6.6 ± 1.9	8.6 ± 3.1	0.38
C3	1.0 ± 0.2	1.0 ± 0.3	1.00	38.5 ± 2.8	41.7 ± 4.8	0.63	27.2 ± 3.0	25.4 ± 1.5	0.63	16.7 ± 1.8	16.2 ± 2.1	1.00	10.8 ± 2.0	9.7 ± 1.1	0.75	5.7 ± 1.2	5.9 ± 1.0	1.00
C4	1.4 ± 0.3	1.1 ± 0.2	1.00	40.9 ± 2.9	38.2 ± 5.5	0.81	28.8 ± 1.7	27.5 ± 3.6	1.00	16.1 ± 2.3	15.0 ± 1.4	1.00	8.3 ± 1.2	11.1 ± 2.0	0.13	4.5 ± 1.0	7.1 ± 1.6	0.13
T5	1.3 ± 0.2	0.6 ± 0.2	0.25	37.7 ± 5.5	32.9 ± 6.5	0.44	22.2 ± 2.2	26.7 ± 0.9	0.13	19.9 ± 3.5	17.1 ± 2.7	0.19	12.5 ± 1.8	13.3 ± 2.4	0.81	6.5 ± 0.9	9.5 ± 2.4	0.25
T6	0.5 ± 0.1	0.6 ± 0.1	0.50	28.0 ± 4.9	35.1 ± 5.0	0.13	28.2 ± 2.5	28.3 ± 0.7	1.00	20.9 ± 2.2	18.8 ± 2.9	0.63	14.5 ± 4.0	11.2 ± 2.7	0.63	7.8 ± 2.6	6.1 ± 1.0	1.00
O1	1.2 ± 0.4	0.4 ± 0.1	0.25	30.5 ± 6.6	30.6 ± 6.7	1.00	23.3 ± 2.0	23.7 ± 2.9	0.81	21.3 ± 2.4	18.9 ± 3.3	0.81	15.5 ± 3.2	15.3 ± 3.5	0.81	8.3 ± 2.1	11.1 ± 3.4	0.38
O2	0.9 ± 0.2	1.2 ± 0.4	0.63	29.3 ± 4.3	40.8 ± 7.9	0.31	25.6 ± 3.7	23.5 ± 1.2	0.63	20.9 ± 2.6	15.6 ± 2.8	0.19	14.9 ± 2.4	11.9 ± 3.5	0.31	8.3 ± 1.6	6.9 ± 1.9	0.63
	**Delta**			**Theta**			**Alpha**			**Beta1**			**Beta2**			**Gamma**		
**3-Back**	**High Score**	**Low Score**	***p***	**High Score**	**Low Score**	***p***	**High Score**	**Low Score**	***p***	**High Score**	**Low Score**	***p***	**High Score**	**Low Score**	***p***	**High Score**	**Low Score**	***p***
Fp1	1.7 ± 0.3	1.4 ± 0.4	1.00	39.8 ± 6.2	39.0 ± 3.8	0.81	24.4 ± 2.3	22.7 ± 1.3	0.81	16.7 ± 2.1	16.4 ± 2.5	1.00	11.8 ± 3.6	14.0 ± 2.1	0.63	5.6 ± 1.2	6.6 ± 0.8	0.63
Fp2	1.9 ± 0.4	1.5 ± 0.5	0.88	36.1 ± 6.4	39.6 ± 4.4	0.81	27.1 ± 4.8	23.7 ± 2.8	0.63	16.0 ± 2.9	14.1 ± 1.5	0.88	11.8 ± 3.0	12.7 ± 3.1	1.00	7.0 ± 1.6	8.5 ± 2.1	0.88
C3	2.3 ± 0.6	1.0 ± 0.1	0.25	43.2 ± 3.9	42.6 ± 3.8	0.63	26.9 ± 1.9	24.6 ± 1.2	0.81	15.6 ± 2.1	15.4 ± 1.0	0.88	8.0 ± 0.9	10.3 ± 1.5	0.44	4.0 ± 0.5	6.1 ± 0.6	0.13
C4	1.6 ± 0.1	1.0 ± 0.2	0.25	35.7 ± 4.6	40.4 ± 3.3	0.88	28.5 ± 2.4	25.5 ± 1.3	0.44	18.4 ± 2.2	17.3 ± 1.3	1.00	10.6 ± 2.1	10.1 ± 1.2	0.81	5.4 ± 0.4	5.7 ± 0.9	0.88
T5	2.0 ± 0.3	0.7 ± 0.1	0.06	42.1 ± 5.2	33.9 ± 3.4	0.63	22.6 ± 3.0	24.7 ± 2.4	0.63	16.7 ± 2.4	17.8 ± 2.0	1.00	10.8 ± 2.3	14.0 ± 1.9	0.44	5.8 ± 0.7	8.9 ± 1.4	0.25
T6	1.0 ± 0.2	0.6 ± 0.1	1.00	28.0 ± 4.9	30.0 ± 5.9	1.00	27.7 ± 2.4	25.8 ± 2.2	0.63	19.4 ± 2.0	19.4 ± 2.2	1.00	16.1 ± 3.9	15.7 ± 3.5	1.00	7.8 ± 2.4	8.5 ± 2.0	1.00
O1	1.0 ± 0.2	0.7 ± 0.2	0.63	30.2 ± 4.3	25.5 ± 5.0	0.63	24.5 ± 4.2	25.6 ± 4.4	0.81	20.1 ± 1.9	23.5 ± 2.6	0.63	14.2 ± 2.7	15.8 ± 4.9	0.81	9.9 ± 2.8	9.0 ± 3.3	1.00
O2	1.1 ± 0.4	0.8 ± 0.2	0.88	29.0 ± 7.0	34.9 ± 3.7	0.44	24.7 ± 1.0	26.7 ± 3.2	0.44	20.3 ± 3.9	18.1 ± 2.5	1.00	16.1 ± 4.3	11.6 ± 2.1	0.63	8.8 ± 2.5	7.9 ± 1.7	0.81

All *p*-values were calculated using the Wilcoxon signed-rank test.

**Table 4:** LOOCV classification accuracy of a.m. vs. p.m. sessions in 2- and 3-back tasks using relative power and raw waveforms.

a.m. vs. p.m. of 2-Back Task	Accuracy Rate of Relative Power	Accuracy Rate of Raw Waveform	a.m. vs. p.m. of 3-Back Task	Accuracy Rate of Relative Power	Accuracy Rate of Raw Waveform
**Participant 1**	41.3 ± 3.5%	60.0 ± 1.8%	Participant 1	44.7 ± 1.4%	51.7 ± 1.6%
**Participant 2**	53.4 ± 1.1%	34.8 ± 1.9%	Participant 2	37.2 ± 1.9%	42.1 ± 1.5%
**Participant 3**	56.4 ± 2.4%	28.9 ± 2.0%	Participant 3	66.6 ± 2.8%	43.7 ± 1.4%
**Participant 4**	57.9 ± 1.3%	51.1 ± 2.6%	Participant 4	54.6 ± 3.0%	59.6 ± 0.9%
**Participant 5**	44.2 ± 1.5%	18.6 ± 1.8%	Participant 5	36.1 ± 1.9%	42.7 ± 1.8%
**Mean**	50.7 ± 3.3%	47.8 ± 5.7%	Mean	38.7 ± 7.5%	48.0 ± 3.4%

**Table 5:** LOOCV classification accuracy of high- vs. low-score sessions in 2- and 3-back tasks using relative power and raw waveforms.

High- vs. Low-Score of 2-Back Task	Accuracy Rate of Relative Power	Accuracy Rate of Raw Waveform	High- vs. Low-Score of 3-Back Task	Accuracy Rate of Relative Power	Accuracy Rate of Raw Waveform
**Participant 1**	52.4 ± 1.6%	57.4 ± 2.2%	Participant 1	53.7 ± 1.6%	41.1 ± 1.7%
**Participant 2**	43.8 ± 2.0%	33.9 ± 1.1%	Participant 2	34.1 ± 2.5%	45.9 ± 1.6%
**Participant 3**	61.2 ± 2.5%	46.9 ± 2.5%	Participant 3	48.1 ± 1.2%	44.1 ± 2.6%
**Participant 4**	27.9 ± 1.2%	63.7 ± 1.6%	Participant 4	44.6 ± 1.8%	46.0 ± 2.0%
**Participant 5**	46.3 ± 1.7%	39.7 ± 1.4%	Participant 5	32.0 ± 2.1%	36.8 ± 3.3%
**Mean**	46.3 ± 5.5%	42.5 ± 4.1%	Mean	48.3 ± 5.5%	42.8 ± 1.7%

## Data Availability

The data presented in this study are available upon request from the corresponding author.

## Abbreviations

The following abbreviations are used in this manuscript:

CNN	Convolutional neural network
EEG	Electroencephalogram
NN	Neural network
WM	Working memory
